# Food-Derived Omega-3 Fatty Acids and Cognitive Aging: Integrating Nutritional Neuroscience and Geroscience

**DOI:** 10.3390/nu18162594

**Published:** 2026-08-07

**Authors:** Noémi Mózes, Ágnes Lipécz, Tamás Csípő, Vince Fazekas-Pongor, Dávid Major, Wei Yi Hung, Virág Zábó, Boglárka Csík, Ágnes Fehér, Bálint Bérczi, Lilla Klesch, Mónika Fekete

**Affiliations:** 1 Institute of Preventive Medicine and Public Health, Faculty of Medicine, Semmelweis University, 1089 Budapest, Hungary; mozes.noemi@semmelweis.hu (N.M.); lipecz.agnes@semmelweis.hu (Á.L.); csipo.tamas@semmelweis.hu (T.C.); pongor.vince@semmelweis.hu (V.F.-P.); major.david@semmelweis.hu (D.M.); hung.wei.yi@semmelweis.hu (W.Y.H.); zabo.virag@semmelweis.hu (V.Z.); csik.boglarka@semmelweis.hu (B.C.); trombitasne.feher.agnes@semmelweis.hu (Á.F.); 2 Health Sciences Division, Doctoral College, Semmelweis University, 1091 Budapest, Hungary; 3 Fodor Center for Prevention and Healthy Aging, Semmelweis University, 1085 Budapest, Hungary; 4 Department of Public Health Medicine, Medical School, University of Pécs, 7624 Pécs, Hungary; berczi.balint84@gmail.com; 5 Infectious Disease Control Department, Beth Israel Deaconess Medical Center, Boston, MA 02215, USA; lilla.klesch04@gmail.com

**Keywords:** omega-3 fatty acids, DHA, EPA, cognitive aging, geroscience, inflammaging, neuroinflammation, brain aging, nutritional neuroscience, healthy aging

## Abstract

Within the emerging field of nutritional neuroscience, omega-3 fatty acids are among the most extensively investigated dietary bioactive compounds with potential relevance to cognitive aging. As populations continue to age worldwide, identifying modifiable nutritional factors that support cognitive health has become an important public health priority. Docosahexaenoic acid (DHA) and eicosapentaenoic acid (EPA), obtained primarily from marine sources, are integral components of neuronal membranes and serve as precursors of specialized pro-resolving lipid mediators. This narrative review integrates concepts from nutritional neuroscience and geroscience to examine how dietary omega-3 fatty acids may influence biological pathways involved in age-related cognitive decline. Current evidence suggests that DHA and EPA affect multiple processes linked to brain aging, including neuroinflammation, synaptic plasticity, mitochondrial function, oxidative stress, cerebrovascular integrity, cellular senescence, and gut–brain axis signaling. These mechanisms overlap with several hallmarks of aging and may contribute to the maintenance of cognitive function during later life. Observational studies generally associate higher dietary intake or circulating omega-3 status with better cognitive performance and a lower risk of cognitive decline and dementia. Findings from randomized controlled trials are less consistent, although benefits appear more likely in individuals with low baseline omega-3 status, mild cognitive impairment, or increased biological vulnerability. Beyond their established anti-inflammatory effects, omega-3 fatty acids may influence multiple aging-related pathways, particularly inflammaging, vascular aging, and mitochondrial dysfunction. Although heterogeneity in study design, dosage, intervention timing, and participant characteristics precludes firm conclusions, viewing omega-3 fatty acids through a geroscience lens offers a useful framework for interpreting existing evidence and informing future research. Overall, omega-3 fatty acids represent a promising nutritional strategy for supporting cognitive health across the lifespan, warranting further investigation in well-characterized populations and precision nutrition approaches.

## 1. Introduction

### 1.1. Population Aging and Cognitive Decline

Population aging has emerged as one of the most significant demographic transformations of the twenty-first century, with profound implications for public health systems worldwide [1]. Improvements in healthcare, sanitation, and socioeconomic conditions have substantially increased life expectancy, resulting in a rapidly expanding proportion of older adults in both developed and developing countries [2]. As longevity increases, age-related chronic diseases, particularly neurodegenerative disorders and cognitive impairment, are becoming increasingly prevalent [3]. Cognitive decline is now recognized as a major contributor to disability, dependency, and reduced quality of life among older adults [4,5].

Dementia represents one of the greatest global health challenges associated with aging populations [6]. According to the World Health Organization, approximately 57 million people worldwide were living with dementia in 2021, with nearly 10 million new cases diagnosed annually. Furthermore, dementia is currently among the leading causes of disability and dependency in older individuals and imposes substantial social and economic burdens on patients, caregivers, and healthcare systems. Alzheimer’s disease accounts for approximately 60–70% of dementia cases, although vascular and mixed pathologies are also highly prevalent in aging populations [7].

Mild cognitive impairment (MCI) has gained increasing attention as an intermediate and potentially transitional stage between normal cognitive aging and dementia [8]. Individuals with MCI exhibit measurable cognitive decline while generally maintaining independence in daily functioning [9]. Importantly, MCI is associated with an elevated risk of progression to dementia, although trajectories may vary considerably between individuals. Recent meta-analyses estimate the global prevalence of MCI among adults aged 50 years and older to range between approximately 15% and 20%, highlighting its major public health relevance [10,11].

Beyond traditional cognitive disorders, the concept of cognitive frailty has emerged as an important geriatric syndrome integrating both physical frailty and cognitive vulnerability in the absence of overt dementia [12]. Cognitive frailty reflects the multidimensional nature of aging and is increasingly recognized within geroscience frameworks linking systemic aging processes to functional decline [13]. This syndrome is associated with increased risks of disability, hospitalization, dementia, and mortality, emphasizing the interconnectedness of physical and cognitive aging mechanisms [13,14].

Given the limited disease-modifying therapies currently available for dementia and related neurodegenerative disorders, preventive strategies targeting modifiable risk factors have become critically important [15]. Increasing evidence suggests that lifestyle-related factors, including diet, physical activity, vascular health, sleep quality, and social engagement, substantially influence cognitive trajectories during aging [16,17]. Consequently, nutritional interventions and dietary bioactive compounds have attracted growing interest as potential tools for promoting healthy brain aging and cognitive resilience [17,18].

### 1.2. Nutrition and Brain Aging

Nutrition has emerged as an important modifiable determinant of healthy cognitive aging. Increasing evidence suggests that dietary habits influence not only cardiometabolic health but also structural and functional aspects of the aging brain [19,20,21,22,23]. This has contributed to the development of nutritional neuroscience, an interdisciplinary field examining the relationship between diet, cognition, and neurodegenerative processes [24,25,26]. Given the lack of effective disease-modifying therapies for dementia, preventive nutritional strategies have gained growing scientific interest [18,27].

Several biological pathways may explain the relationship between diet and cognitive aging. Dietary factors can modulate systemic inflammation, oxidative stress, mitochondrial function, endothelial health, insulin sensitivity, and gut microbiota composition, all of which are increasingly recognized as contributors to brain aging and neurodegeneration [20,28,29,30,31,32]. Importantly, many of these mechanisms overlap substantially with the hallmarks of aging described within geroscience frameworks, including inflammaging, mitochondrial dysfunction, altered intercellular communication, and impaired nutrient sensing [33]. Consequently, dietary interventions may represent promising non-pharmacological approaches to support cognitive healthspan and resilience during aging [20,34].

Among dietary patterns, the Mediterranean diet has received particularly strong attention in relation to cognitive health. Characterized by high consumption of fruits, vegetables, legumes, whole grains, olive oil, nuts, and fish, alongside low intake of processed foods and saturated fats, the Mediterranean diet has consistently been associated with better cognitive performance and reduced risk of cognitive decline and dementia in observational studies [22,34,35,36,37]. Potential mechanisms include anti-inflammatory and antioxidant effects, improved vascular function, and favorable modulation of metabolic health [38]. Moreover, adherence to Mediterranean-style dietary patterns has been linked to slower brain atrophy and improved cognitive trajectories in older adults [39].

Importantly, contemporary nutritional research increasingly emphasizes overall dietary patterns rather than isolated nutrients alone. Although individual bioactive compounds, including omega-3 fatty acids, polyphenols, and vitamins, may exert neuroprotective effects, foods are consumed within complex dietary matrices characterized by synergistic interactions between nutrients [40,41]. Dietary pattern approaches may therefore provide a more physiologically relevant understanding of how nutrition influences cognitive aging [42]. Nevertheless, investigating specific nutrients remains valuable for identifying mechanistic pathways and potential therapeutic targets. In this context, omega-3 fatty acids have emerged as particularly relevant candidates due to their structural and functional roles in neuronal membranes, inflammation resolution, and cerebrovascular regulation [43].

### 1.3. Aim of the Review

Given the growing burden of cognitive decline and the limited availability of effective disease-modifying therapies, identifying modifiable factors that support healthy brain aging has become increasingly important. Among nutritional factors, omega-3 fatty acids have attracted considerable interest due to their structural, anti-inflammatory, and neuroprotective properties [43,44].

This narrative review aims to integrate evidence from nutritional neuroscience and geroscience to examine how food-derived omega-3 fatty acids may influence biological mechanisms involved in cognitive aging. We summarize current mechanistic, epidemiological, and clinical evidence regarding their roles in neuroinflammation, synaptic plasticity, mitochondrial function, oxidative stress, and cerebrovascular health, while highlighting the potential relevance of geroscience-based approaches for promoting cognitive resilience and healthy brain aging.

## 2. Methods

A structured narrative literature review was conducted to identify relevant publications examining omega-3 fatty acids, cognitive aging, brain aging, and geroscience-related mechanisms. Searches were performed in PubMed, Scopus, and Web of Science for studies published up to December 2025. Search strategies combined keywords and Medical Subject Headings (MeSH) terms including “omega-3 fatty acids”, “DHA”, “EPA”, “brain aging”, “cognitive aging”, “cognitive decline”, “dementia”, “geroscience”, “inflammaging”, “neuroinflammation”, “mitochondrial dysfunction”, and “vascular aging” using Boolean operators (AND/OR).

Peer-reviewed studies published in English were considered eligible. Priority was given to high-quality evidence, including systematic reviews, meta-analyses, randomized controlled trials, large prospective cohort studies, and landmark experimental investigations relevant to cognitive aging and neurodegeneration. Mechanistic in vitro and in vivo studies were also included to provide biological context regarding the effects of omega-3 fatty acids on aging-related pathways.

Studies were selected based on scientific relevance, methodological quality, and relevance to the conceptual framework of geroscience and nutritional neuroscience. Particular emphasis was placed on publications addressing neuroinflammation, synaptic plasticity, mitochondrial function, oxidative stress, cerebrovascular integrity, and cognitive outcomes associated with aging. Reference lists of key articles and relevant reviews were additionally screened to identify further eligible studies. Although this work is a narrative review rather than a formal systematic review, structured search and selection principles were applied to enhance transparency, scientific rigor, and reproducibility. No formal systematic review protocol or meta-analytic methodology was employed.

## 3. Biology and Metabolism of Omega-3 Fatty Acids

Omega-3 fatty acids are essential polyunsaturated fatty acids with important structural, metabolic, and signaling functions in human physiology, particularly within the nervous system [45]. Given their involvement in membrane composition, inflammatory regulation, and neuronal function, understanding their biological characteristics and metabolism is important before discussing their potential relevance to cognitive aging and geroscience-related mechanisms [46].

### 3.1. Types and Dietary Sources of Omega-3 Fatty Acids

The principal omega-3 fatty acids relevant to human health include alpha-linolenic acid (ALA), eicosapentaenoic acid (EPA), and docosahexaenoic acid (DHA) [47,48]. ALA is an essential fatty acid that cannot be synthesized endogenously and must therefore be obtained through dietary intake. Major plant-derived sources of ALA include flaxseed, chia seeds, walnuts, hemp seeds, and certain vegetable oils such as canola and soybean oil [47,49,50].

In contrast, EPA and DHA are predominantly found in marine sources, including fatty fish such as salmon, mackerel, sardines, herring, and tuna [51,52]. Marine algae represent the primary biological producers of DHA and EPA within aquatic ecosystems and have also emerged as important alternative sources of omega-3 fatty acids, particularly for vegetarian and vegan dietary patterns. Algae-derived supplements may therefore provide a sustainable non-fish source of long-chain omega-3 fatty acids [53,54,55,56].

Although humans can convert ALA into EPA and DHA through a series of elongation and desaturation reactions, the efficiency of this conversion is relatively low, particularly for DHA synthesis [57]. Consequently, direct dietary intake of EPA and DHA is generally considered more effective for increasing tissue concentrations of long-chain omega-3 fatty acids [58].

DHA is highly enriched in neuronal membranes and represents one of the most abundant polyunsaturated fatty acids in the brain, where it contributes to membrane fluidity, synaptic signaling, and neuroplasticity [19,59,60]. EPA, while present at lower concentrations in neural tissue, exerts important anti-inflammatory and vascular regulatory effects that may indirectly support cognitive function and healthy brain aging [43,61,62].

In addition to dietary intake through whole foods, omega-3 fatty acids are widely available as nutritional supplements, including fish oil, krill oil, and algae-derived formulations [63,64]. However, variability in dosage, bioavailability, EPA:DHA composition, and formulation characteristics contributes to heterogeneity across clinical studies evaluating cognitive outcomes [65].

### 3.2. Absorption, Transport, and Brain Incorporation

#### 3.2.1. Intestinal Digestion and Absorption of Omega-3 Fatty Acids

Following dietary intake, omega-3 fatty acids undergo intestinal digestion and absorption primarily in the small intestine. Dietary triglycerides containing DHA and EPA are hydrolyzed by pancreatic lipases into free fatty acids and monoacylglycerols, which are subsequently incorporated into mixed micelles together with bile salts [45,66]. These micelles facilitate uptake into enterocytes, where omega-3 fatty acids are re-esterified into triglycerides and phospholipids before being packaged into chylomicrons for systemic transport via the lymphatic circulation [67].

#### 3.2.2. Systemic Transport and Factors Influencing Bioavailability

After entering the bloodstream, omega-3 fatty acids are transported in association with circulating lipoproteins, including chylomicrons, very-low-density lipoproteins (VLDL), low-density lipoproteins (LDL), and high-density lipoproteins (HDL) [68]. In plasma, DHA and EPA may also circulate as non-esterified fatty acids bound to albumin or incorporated into phospholipid fractions [69]. The bioavailability and tissue distribution of omega-3 fatty acids are influenced by several factors, including food matrix, lipid composition, genetic background, age, metabolic health, and formulation characteristics of supplements [70,71].

#### 3.2.3. Blood–Brain Barrier Transport Mechanisms

Transport of omega-3 fatty acids across the blood–brain barrier (BBB) is a critical step for maintaining cerebral lipid homeostasis and neuronal function. Although passive diffusion may contribute to limited uptake, growing evidence suggests that specific transport mechanisms play major roles in brain delivery of DHA [72]. In particular, the major facilitator superfamily domain-containing protein 2A (MFSD2A) has been identified as an important transporter mediating uptake of lysophosphatidylcholine (LPC)-bound DHA across the BBB [73]. Impairment of DHA transport mechanisms has been linked to neurodevelopmental abnormalities and may also contribute to age-related cognitive decline and neurodegeneration [73].

#### 3.2.4. Brain Incorporation and Functional Roles of DHA and EPA

Within the brain, DHA becomes highly enriched in neuronal membrane phospholipids, especially in synaptic membranes, mitochondria, and photoreceptor cells [59]. Membrane incorporation of DHA influences membrane fluidity, receptor function, neurotransmission, ion channel activity, and synaptic plasticity [74]. These properties are considered particularly important for learning, memory, and cognitive resilience during aging. EPA is present at substantially lower concentrations in the brain compared to DHA; however, it may exert indirect neuroprotective effects through modulation of systemic inflammation, endothelial function, and neuroimmune signaling [75].

#### 3.2.5. Age-Related Changes in Omega-3 Metabolism and Brain Availability

Importantly, aging itself may alter omega-3 fatty acid metabolism and cerebral delivery. Age-related changes in intestinal absorption, hepatic metabolism, lipoprotein dynamics, and blood–brain barrier integrity may influence brain omega-3 availability in older adults [76]. Furthermore, metabolic disorders commonly associated with aging, including obesity, insulin resistance, and cardiovascular disease, may impair omega-3 transport and utilization [77]. These factors may partially explain heterogeneity observed across clinical studies investigating omega-3 supplementation and cognitive outcomes in aging populations.

### 3.3. Specialized Pro-Resolving Mediators

#### 3.3.1. Formation and Biological Significance of Specialized Pro-Resolving Mediators

Beyond their structural roles in cellular membranes, omega-3 fatty acids serve as precursors for a family of bioactive lipid mediators collectively known as specialized pro-resolving mediators (SPMs), which include resolvins, protectins, and maresins [78]. These molecules are generated primarily from EPA and DHA through enzymatic pathways involving cyclooxygenases and lipoxygenases and play critical roles in the active resolution of inflammation [79].

#### 3.3.2. Resolvins and Regulation of Neuroinflammation

Resolvins are subdivided into E-series resolvins derived from EPA and D-series resolvins derived from DHA. These mediators regulate inflammatory responses by reducing leukocyte infiltration, suppressing pro-inflammatory cytokine production, and promoting clearance of cellular debris and apoptotic cells [80]. Experimental studies suggest that resolvins may also modulate microglial activation and neuroimmune signaling within the central nervous system, thereby contributing to neuroprotection during aging and neurodegeneration [75].

#### 3.3.3. Protectins and the Neuroprotective Role of Neuroprotectin D1

Protectins, particularly neuroprotectin D1 (NPD1), are DHA-derived mediators with important anti-inflammatory and neuroprotective properties [81]. NPD1 has been shown to regulate oxidative stress responses, inhibit neuronal apoptosis, and attenuate amyloid-beta-induced neurotoxicity in experimental models of Alzheimer’s disease [82]. Reduced levels of neuroprotective lipid mediators have been observed in aging and neurodegenerative conditions, suggesting impaired inflammation-resolution pathways may contribute to cognitive decline [83].

#### 3.3.4. Maresins in Tissue Repair and Brain Homeostasis

Maresins, another class of DHA-derived mediators synthesized primarily by macrophages, are increasingly recognized for their roles in tissue repair, immune regulation, and restoration of homeostasis following inflammatory stress [84,85]. Emerging evidence indicates that maresins may support blood–brain barrier integrity, reduce neuroinflammation, and promote neuronal survival, although their specific relevance to cognitive aging remains under active investigation [86].

#### 3.3.5. Specialized Pro-Resolving Mediators in Aging and Cognitive Decline

**Figure 1:**
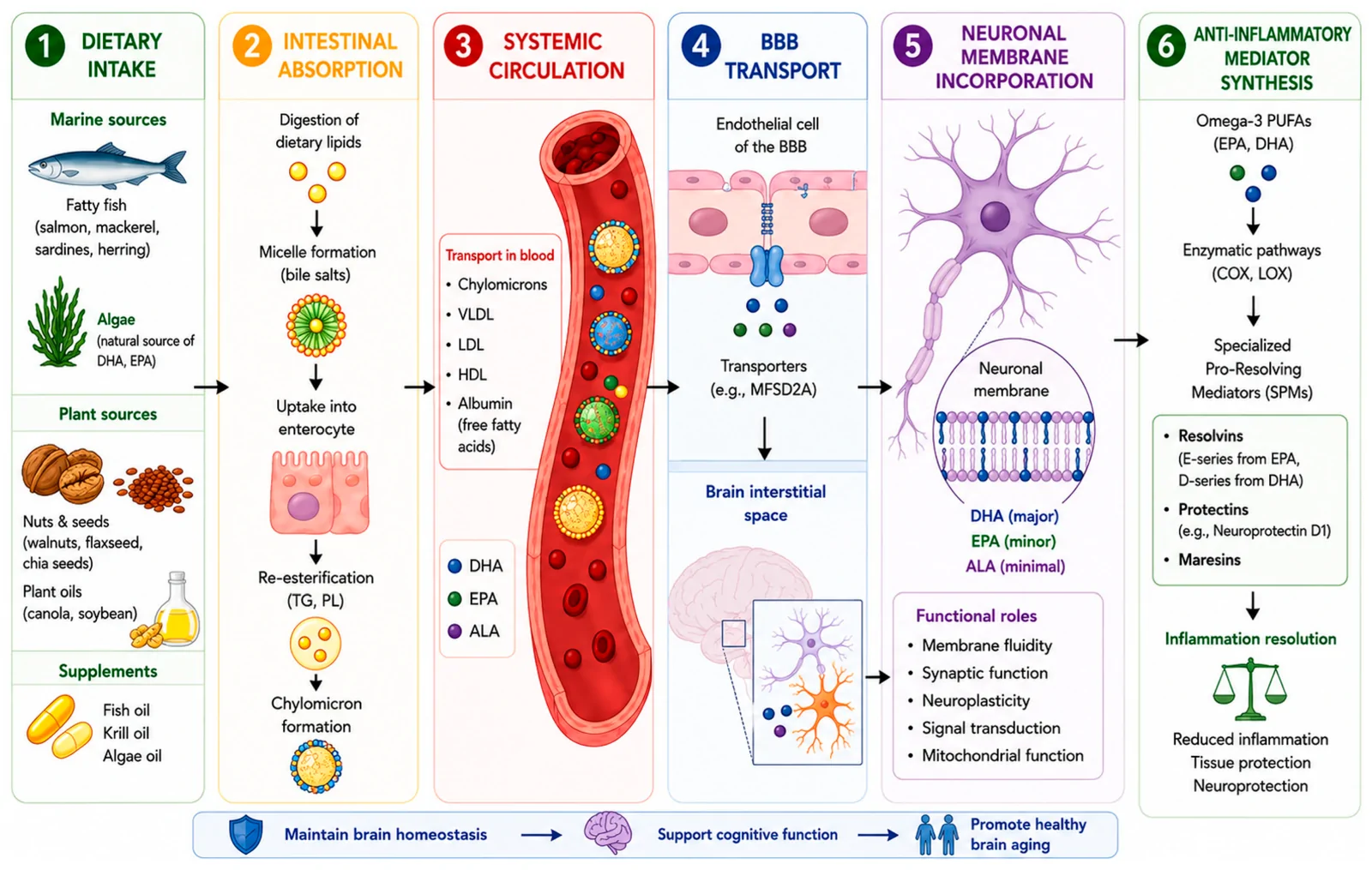
Overview of omega-3 fatty acid metabolism and brain incorporation. Dietary omega-3 fatty acids derived from marine, plant, algal, and supplemental sources undergo intestinal absorption and systemic transport before crossing the blood–brain barrier and becoming incorporated into neuronal membranes. Within the brain, DHA contributes to multiple aspects of neuronal function and serves as a precursor for specialized pro-resolving mediators (SPMs), including resolvins, protectins, and maresins, which support inflammation resolution, tissue homeostasis, and neuroprotection [45,59,66,67,68,69,70,71,72,73,74,75,76,77,78,79,80,81,82,83,84,85,86]. Abbreviations: ALA, alpha-linolenic acid; BBB, blood–brain barrier; DHA, docosahexaenoic acid; EPA, eicosapentaenoic acid; HDL, high-density lipoprotein; LDL, low-density lipoprotein; LPC, lysophosphatidylcholine; MFSD2A, major facilitator superfamily domain-containing protein 2A; PL, phospholipids; SPMs, specialized pro-resolving mediators; TG, triglycerides; VLDL, very-low-density lipoprotein. Color coding: DHA is shown in blue, EPA in green, and ALA in purple.

Importantly, the resolution of inflammation is now recognized as an active and tightly regulated biological process rather than a passive termination of the inflammatory response [79]. Chronic low-grade inflammation, commonly referred to as inflammaging, is a central hallmark of biological aging and has been strongly implicated in neurodegeneration and cognitive decline [83]. Accordingly, impaired biosynthesis or biological activity of SPMs may contribute to age-related inflammatory dysregulation, impaired tissue homeostasis, and reduced cognitive resilience. Dietary omega-3 fatty acids, including ALA, EPA, and DHA, serve as substrates for cyclooxygenase (COX), lipoxygenase (LOX), and cytochrome P450 (CYP) enzymes, leading to the biosynthesis of SPMs, including resolvins, protectins, and maresins [78,79,80,81]. Unlike conventional anti-inflammatory agents, which primarily suppress inflammatory signaling, these endogenous lipid mediators actively orchestrate the resolution phase of inflammation by reducing pro-inflammatory cytokine production, limiting excessive microglial activation, promoting efferocytosis and tissue repair, attenuating oxidative stress, and restoring tissue homeostasis [79,80,81,82]. Within the central nervous system, these coordinated actions may reduce chronic neuroinflammation, preserve synaptic integrity, and enhance neuronal resilience, thereby slowing cognitive decline associated with biological aging and neurodegenerative disorders [83,84,85,86,87]. An overview of dietary sources, systemic transport, brain incorporation, and downstream conversion of omega-3 fatty acids into specialized pro-resolving mediators is presented in Figure 1.

## 4. Mechanisms Linking Omega-3 Fatty Acids to Cognitive Aging

### 4.1. Neuroinflammation and Inflammaging

Chronic low-grade inflammation is a hallmark of biological aging and is increasingly recognized as a central contributor to cognitive decline and neurodegeneration [88,89]. Aging is associated with progressive dysregulation of innate and adaptive immune responses, a phenomenon often referred to as “inflammaging”, characterized by persistently elevated levels of pro-inflammatory mediators including interleukin-6 (IL-6), tumor necrosis factor-alpha (TNF-α), and C-reactive protein (CRP) [89,90]. These inflammatory processes may contribute to synaptic dysfunction, neuronal injury, impaired neurogenesis, and accelerated neurodegenerative pathology.

Within the central nervous system, microglia play a critical role in age-related neuroinflammation. Under physiological conditions, microglia contribute to immune surveillance, synaptic remodeling, and tissue homeostasis. However, aging promotes a shift toward chronic microglial activation, resulting in excessive production of pro-inflammatory cytokines, reactive oxygen species, and neurotoxic mediators [91,92]. Sustained microglial activation has been implicated in the pathogenesis of Alzheimer’s disease and other neurodegenerative disorders associated with cognitive impairment [93].

Omega-3 fatty acids, particularly DHA and EPA, exert multiple anti-inflammatory effects that may counteract these aging-related inflammatory pathways [44]. Experimental studies have demonstrated that omega-3 fatty acids can suppress nuclear factor-kappa B (NF-κB) signaling, reduce production of IL-6 and TNF-α, and modulate microglial activation states toward more neuroprotective phenotypes [75,94]. In addition, omega-3 fatty acids influence membrane lipid composition and receptor signaling, thereby affecting immune cell communication and inflammatory responsiveness [74].

Importantly, DHA and EPA also serve as precursors for specialized pro-resolving mediators, including resolvins, protectins, and maresins, which actively promote the resolution of inflammation [79,95]. These mediators facilitate clearance of inflammatory debris, attenuate cytokine production, and support restoration of tissue homeostasis. Emerging evidence suggests that impaired inflammation-resolution pathways may contribute to persistent neuroinflammation during aging and neurodegeneration [37].

### 4.2. Synaptic Plasticity and Neurotransmission

Synaptic plasticity, defined as the ability of neuronal circuits to modify their structure and function in response to experience, is fundamental for learning, memory formation, and cognitive adaptability throughout life. Aging is associated with progressive impairments in synaptic plasticity, including reduced synaptic density, altered neurotransmitter signaling, and diminished capacity for long-term potentiation (LTP), particularly within the hippocampus, a brain region critically involved in memory processing [96,97]. These alterations are thought to contribute substantially to age-related cognitive decline and increased vulnerability to neurodegenerative disorders.

Omega-3 fatty acids, especially DHA, are highly enriched in neuronal membranes and play essential roles in maintaining membrane structure and function. Due to its unique biophysical properties, DHA enhances membrane fluidity, influences lipid raft organization, and modulates the activity of membrane-bound receptors, ion channels, and signaling proteins [43,74]. Through these mechanisms, omega-3 fatty acids can affect synaptic transmission and neuronal communication, thereby supporting efficient information processing within neural networks.

Accumulating evidence further suggests that omega-3 fatty acids regulate key molecular pathways involved in neuroplasticity. In particular, DHA and EPA have been associated with increased expression of brain-derived neurotrophic factor (BDNF), a neurotrophin that promotes neuronal survival, dendritic growth, synaptogenesis, and synaptic remodeling [19,98]. Reduced BDNF signaling has been linked to cognitive impairment, neurodegeneration, and age-related hippocampal dysfunction, whereas interventions that enhance BDNF activity are generally associated with improved cognitive performance and neural resilience [99].

Experimental and clinical studies indicate that omega-3 fatty acid intake may support hippocampal integrity and cognitive function through effects on synaptic plasticity, neurogenesis, and neurotransmitter systems [100]. Animal models have demonstrated improvements in learning and memory performance following DHA supplementation, while human studies suggest associations between higher omega-3 status and better cognitive outcomes, particularly in older adults at risk of cognitive decline [75,101]. From a geroscience perspective, preservation of synaptic function may represent an important mechanism through which nutritional interventions contribute to maintaining cognitive resilience and extending brain healthspan during aging [102,103,104,105,106,107,108,109,110,111,112,113,114,115,116].

### 4.3. Mitochondrial Dysfunction and Oxidative Stress

Mitochondrial dysfunction is a fundamental hallmark of biological aging and plays a central role in age-related cognitive decline and neurodegenerative diseases [117,118]. Neurons are particularly vulnerable to mitochondrial impairment because of their high energy demands and dependence on oxidative phosphorylation for adenosine triphosphate (ATP) production. Aging is associated with progressive declines in mitochondrial efficiency, impaired electron transport chain activity, reduced ATP generation, and increased production of reactive oxygen species (ROS), which collectively contribute to neuronal dysfunction and cognitive deterioration [117,119].

Excessive ROS generation promotes oxidative damage to lipids, proteins, and nucleic acids, thereby disrupting cellular homeostasis and accelerating neurodegenerative processes [120]. The aging brain is especially susceptible to oxidative stress due to its high oxygen consumption, abundant lipid content, and relatively limited antioxidant capacity. Accumulation of oxidative damage has been linked to synaptic dysfunction, impaired neurogenesis, mitochondrial DNA mutations, and progressive cognitive decline [121].

Emerging evidence suggests that omega-3 fatty acids may mitigate several aspects of mitochondrial aging. DHA is highly enriched in mitochondrial membranes, where it contributes to membrane fluidity, structural integrity, and optimal functioning of membrane-associated proteins [74,122]. Experimental studies indicate that omega-3 fatty acids can improve mitochondrial bioenergetics, enhance respiratory efficiency, and reduce excessive ROS production, thereby supporting neuronal energy metabolism and cellular resilience [123,124,125]. In addition, omega-3 fatty acids may indirectly strengthen endogenous antioxidant defenses through modulation of redox-sensitive signaling pathways, including nuclear factor erythroid 2-related factor 2 (Nrf2) [126].

Beyond their effects on oxidative stress, omega-3 fatty acids may contribute to maintenance of mitochondrial quality control mechanisms, including mitophagy and protein homeostasis pathways. Impaired removal of damaged mitochondria and accumulation of misfolded proteins are increasingly recognized as interconnected drivers of neuronal aging and neurodegeneration [127]. By preserving mitochondrial function and limiting oxidative injury, omega-3 fatty acids may help sustain neuronal viability and cognitive performance during aging.

### 4.4. Cerebrovascular Aging and Endothelial Function

Growing evidence indicates that vascular aging plays a critical role in cognitive decline and dementia. Beyond classical neurodegenerative mechanisms, age-related alterations in the cerebral vasculature substantially contribute to cognitive impairment through reduced cerebral blood flow, impaired neurovascular coupling, and disruption of blood–brain barrier (BBB) function [128,129]. The concept of vascular contributions to cognitive impairment and dementia (VCID) has therefore emerged as an important framework linking cardiovascular and cerebrovascular health to cognitive aging [130].

Aging is associated with progressive endothelial dysfunction characterized by reduced nitric oxide bioavailability, increased oxidative stress, chronic inflammation, and impaired vasodilatory capacity [131]. These changes contribute to arterial stiffness and microvascular dysfunction, both of which are associated with diminished cerebral perfusion and increased risk of cognitive decline [132]. Longitudinal studies have demonstrated that vascular dysfunction often precedes measurable cognitive impairment, suggesting that cerebrovascular aging may represent an early and potentially modifiable driver of neurodegeneration [133].

The integrity of the blood–brain barrier is another critical determinant of brain health during aging. The BBB regulates the exchange of nutrients, metabolites, and signaling molecules between the systemic circulation and the central nervous system. However, aging is associated with increased BBB permeability, endothelial cell dysfunction, and impaired clearance of neurotoxic proteins, including amyloid-β [134,135]. BBB breakdown has been increasingly recognized as an early pathological event contributing to neuroinflammation, neuronal dysfunction, and cognitive decline [136].

Omega-3 fatty acids may exert protective effects on multiple components of the aging cerebrovascular system. Experimental and clinical studies suggest that DHA and EPA improve endothelial function, enhance nitric oxide signaling, reduce vascular inflammation, and attenuate oxidative stress within the vascular wall [44,137,138]. Furthermore, omega-3 fatty acids have been shown to support microvascular function and may contribute to preservation of cerebral blood flow and neurovascular coupling [105]. Emerging evidence also indicates that DHA is important for maintaining BBB integrity through modulation of endothelial membrane composition, inflammatory signaling, and tight junction protein expression [139].

### 4.5. Cellular Senescence and Neurodegeneration

Cellular senescence is increasingly recognized as a fundamental driver of biological aging and age-related diseases, including neurodegenerative disorders [140,141]. Senescent cells undergo irreversible cell-cycle arrest while remaining metabolically active and acquiring a distinctive senescence-associated secretory phenotype (SASP), characterized by the production of pro-inflammatory cytokines, chemokines, growth factors, and matrix-remodeling enzymes [142,143]. Although cellular senescence initially serves protective functions, including tumor suppression and tissue repair, the accumulation of senescent cells during aging may promote chronic inflammation, tissue dysfunction, and impaired regenerative capacity.

In the central nervous system, senescence-like phenotypes have been identified in multiple cell types, including astrocytes, microglia, oligodendrocyte precursor cells, endothelial cells, and, under certain conditions, neurons [144]. Accumulating evidence suggests that SASP factors may contribute to a pro-inflammatory brain microenvironment, thereby amplifying neuroinflammation and disrupting neuronal homeostasis [145]. These mechanisms have attracted considerable attention within geroscience, as cellular senescence may represent a biological link between aging processes and neurodegenerative pathology.

Cellular senescence has also been implicated in the development and progression of Alzheimer’s disease and related neurodegenerative disorders. Experimental studies indicate that senescent glial cells may promote amyloid-beta accumulation, tau hyperphosphorylation, synaptic dysfunction, and neuronal injury through chronic inflammatory signaling and impaired clearance mechanisms [145,146]. Furthermore, postmortem studies have reported increased expression of senescence-associated markers in brain regions affected by neurodegeneration, although causal relationships remain under investigation [147].

Emerging evidence suggests that omega-3 fatty acids may influence pathways associated with cellular senescence and neurodegeneration. Through their anti-inflammatory, antioxidant, and pro-resolving properties, DHA and EPA may attenuate SASP-related signaling and reduce cellular stress responses that contribute to senescence induction [148]. Experimental studies have further demonstrated that omega-3 fatty acids may modulate amyloid-beta metabolism, reduce tau-related pathology, and support neuronal survival, although findings have not been entirely consistent across experimental models and clinical studies [43,149,150,151]. Therefore, while omega-3 fatty acids are unlikely to directly eliminate senescent cells, they may help mitigate downstream consequences of senescence-associated inflammation and cellular dysfunction.

### 4.6. Gut–Brain Axis and Microbiome Interactions

The gut–brain axis has emerged as a complex bidirectional communication network linking the gastrointestinal tract, immune system, metabolism, and central nervous system function [152,153]. Growing evidence suggests that alterations in gut microbiota composition may influence cognitive aging and neurodegenerative processes through multiple interconnected pathways involving neuroinflammation, immune regulation, and metabolic signaling [154,155]. Consequently, the gut microbiome is increasingly recognized as an important contributor to brain health within contemporary geroscience frameworks [156].

Aging is associated with progressive changes in gut microbial composition and diversity, often characterized by reductions in beneficial commensal microorganisms and expansion of pro-inflammatory microbial taxa [157,158]. These alterations, collectively referred to as age-related dysbiosis, may contribute to chronic low-grade systemic inflammation and immune dysfunction, thereby promoting inflammaging and increasing vulnerability to age-related diseases [83]. Furthermore, aging-related disruption of intestinal barrier integrity may facilitate translocation of microbial products, including lipopolysaccharides (LPS), into the circulation, triggering systemic inflammatory responses that can affect the central nervous system [159].

Microbial metabolites serve as key mediators of gut–brain communication [160]. Short-chain fatty acids (SCFAs), particularly acetate, propionate, and butyrate, regulate immune homeostasis, microglial activity, blood–brain barrier integrity, and neuronal signaling pathways [161]. In addition, microbial metabolism of dietary components generates bioactive metabolites capable of influencing neurotransmitter synthesis, neuroplasticity, and brain energy metabolism [153]. Emerging evidence suggests that age-related alterations in microbial metabolite production may contribute to cognitive impairment and neurodegenerative pathology [154,162,163].

Recent studies indicate that omega-3 fatty acids may beneficially modulate the gut microbiome and its metabolic activity [164]. Both experimental and clinical investigations have reported associations between DHA and EPA intake and increased microbial diversity, enrichment of beneficial bacterial taxa, and enhanced production of anti-inflammatory metabolites [165,166]. Omega-3 fatty acids may also strengthen intestinal barrier function and reduce gut-derived inflammation, thereby influencing gut–brain communication pathways relevant to cognitive health [167,168]. Furthermore, interactions between omega-3 fatty acids and the gut microbiome may partially explain interindividual differences observed in cognitive responses to omega-3 supplementation [164,169].

### 4.7. Specialized Pro-Resolving Mediators and Additional Neuroprotective Mechanisms of Omega-3 Fatty Acids

Beyond their structural role in neuronal membranes, omega-3 fatty acids exert important biological effects through their conversion into specialized pro-resolving mediators (SPMs), including resolvins, protectins, and maresins, which actively promote the resolution of inflammation and restoration of tissue homeostasis [78,81]. These mediators modulate microglial activation, reduce pro-inflammatory cytokine production, and support neuronal resilience in experimental models of neurodegeneration [81]. In addition to their pro-resolving actions, omega-3 fatty acids may influence pathological processes associated with Alzheimer’s disease, including amyloid-β metabolism, tau-related pathology, and neuronal survival [82,149,170,171,172]. Experimental evidence further suggests that DHA and EPA may support neuronal resilience through effects on mitochondrial function, neurotrophic signaling, and cellular survival pathways [44]. Collectively, these mechanisms provide a biologically plausible basis for the neuroprotective effects of omega-3 fatty acids, although their translation into consistent clinical benefits remains incompletely established [173,174].

## 5. Evidence from Human Studies

### 5.1. Observational Studies

Observational studies have provided important insights into the potential relationship between omega-3 fatty acid exposure and cognitive aging. Large population-based cohorts have evaluated both dietary intake and circulating biomarkers of omega-3 status in relation to cognitive performance, cognitive decline, and incident dementia [163,173]. Overall, many observational studies suggest that higher omega-3 fatty acid exposure is associated with better cognitive outcomes and a lower risk of age-related cognitive impairment, although findings remain heterogeneous across populations and study designs [175].

Dietary intake studies have frequently reported positive associations between fish consumption and cognitive health. Prospective cohort studies indicate that individuals with higher consumption of fish and seafood generally exhibit slower rates of cognitive decline and lower risks of dementia compared with those reporting lower intake levels [175,176,177,178,179,180]. Similar findings have been observed in cohorts adhering to Mediterranean-style dietary patterns and other brain-healthy diets rich in fish and seafood, suggesting that omega-3 fatty acids may contribute to broader dietary effects on cognitive health and dementia prevention [181,182,183]. However, the relative contribution of omega-3 fatty acids independent of other dietary components remains difficult to determine [184].

Beyond dietary assessment, biomarker-based studies have strengthened the evidence base by reducing measurement error associated with self-reported dietary intake. Plasma, erythrocyte, and phospholipid omega-3 concentrations have been associated with favorable cognitive trajectories and reduced risks of cognitive decline in several longitudinal studies [185,186]. Higher circulating levels of DHA, in particular, have been linked to larger brain volumes, better memory performance, and lower incidence of dementia in older adults [187,188]. Biomarker studies may therefore provide a more objective estimate of long-term omega-3 status than food frequency questionnaires alone [187].

Longitudinal cohort studies have further examined the relationship between omega-3 status and incident dementia. Several large prospective investigations have reported inverse associations between fish consumption, circulating DHA concentrations, or omega-3 indices and the risk of Alzheimer’s disease and all-cause dementia [105,189,190]. Nevertheless, not all studies have demonstrated significant protective associations, and effect sizes are often modest after adjustment for demographic, lifestyle, and vascular risk factors [109,191].

Interpretation of observational findings should be undertaken with caution, as participants with higher omega-3 exposure frequently exhibit more favorable lifestyle and health characteristics than those with lower exposure. Despite statistical adjustment for a wide range of demographic, behavioral, and vascular risk factors, residual confounding remains a potential explanation for part of the observed protective associations [186]. Residual confounding may therefore partly explain observed associations. Reverse causation is also a potential concern, as individuals experiencing early cognitive decline may modify dietary habits before clinical diagnosis [15,16]. Furthermore, omega-3 intake commonly occurs within broader dietary patterns, making it challenging to isolate the specific contribution of individual fatty acids from other beneficial nutritional exposures [182].

Taken together, observational studies generally support an association between higher omega-3 fatty acid exposure and healthier cognitive aging. However, the inherent limitations of observational research preclude causal inference, highlighting the importance of randomized controlled trials to determine whether increasing omega-3 intake directly influences cognitive outcomes in aging populations (Table 1) [15].

### 5.2. Randomized Controlled Trials

Randomized controlled trials investigating omega-3 fatty acid supplementation have generated mixed but suggestive evidence regarding cognitive aging. While observational studies consistently report associations between higher omega-3 status and better cognitive outcomes, intervention studies generally demonstrate modest and population-dependent effects. Differences in DHA/EPA dosage, intervention duration, baseline nutritional status, genetic background, vascular health, and cognitive phenotype likely contribute to the substantial heterogeneity observed across studies [101,173,195,196,197,198,199,200,201,202,203,204,205].

The most promising findings have emerged in individuals with mild cognitive impairment (MCI), a population considered particularly relevant for preventive interventions targeting age-related cognitive decline. Several randomized trials have reported improvements in memory performance, executive function, or biomarkers associated with neurodegeneration following DHA-rich supplementation [196,197,200,203]. In a 12-month placebo-controlled trial, supplementation with 1491 mg/day DHA and 351 mg/day EPA significantly increased omega-3 status, although no significant improvements were observed in global cognitive outcomes. Nevertheless, favorable effects on depressive symptoms and cardiovascular parameters were detected, particularly among APOE ε4 carriers, suggesting potential benefits beyond cognition alone [196]. Similarly, recent evidence indicates that DHA supplementation combined with medium-chain triglycerides may produce greater cognitive improvements than either intervention alone, highlighting the importance of metabolic context and brain energy utilization in determining treatment response [200].

Among cognitively healthy older adults, findings have generally been less consistent. Several long-term supplementation studies have reported no significant improvements in global cognition, episodic memory, or executive function despite substantial increases in circulating DHA and EPA concentrations [101,198,204,205,206,207,208]. Although some trials have demonstrated modest benefits in specific domains, including working memory, attention, processing speed, and verbal fluency, these effects have not been consistently replicated across populations or cognitive assessment methods [202,205,209]. In particular, studies incorporating additional nutrients such as carotenoids and vitamin E have occasionally reported greater cognitive improvements, although the independent contribution of omega-3 fatty acids is difficult to isolate in such multimodal interventions [205].

Intervention dosage and duration appear to be important determinants of efficacy. Most trials have employed daily doses ranging from approximately 500 mg to over 2 g of DHA and EPA, with intervention periods varying between six months and two years. A randomized placebo-controlled trial demonstrated that high-dose DHA supplementation (2.15 g/day) significantly increased cerebrospinal fluid DHA concentrations, confirming effective delivery to the central nervous system [195]. However, despite marked improvements in biological markers, cognitive outcomes remained largely unchanged during the six-month intervention period, illustrating a recurring challenge in omega-3 research whereby favorable biochemical effects do not necessarily translate into short-term cognitive benefits [195].

The frequent occurrence of null findings has prompted considerable interest in identifying responder populations. One prominent explanation is the baseline deficiency hypothesis, which proposes that supplementation may be most effective in individuals with low habitual omega-3 intake or suboptimal omega-3 status [202,204]. In many trials, participants are recruited from relatively well-nourished populations, thereby limiting the potential for measurable improvement. Furthermore, substantial interindividual variability exists with respect to age, APOE genotype, inflammatory burden, cardiometabolic health, cerebrovascular function, and baseline cognitive performance, all of which may influence responsiveness to omega-3 supplementation [196,197,204].

In the context of biological aging, omega-3 fatty acids may exert their greatest effects through modulation of aging-related biological mechanisms rather than through direct enhancement of cognition. Improvements in neuroinflammation, endothelial function, cerebral perfusion, oxidative stress, and metabolic resilience may contribute to the maintenance of cognitive function over time, particularly in individuals exhibiting elevated biological vulnerability [195,197,199,200,201,202]. Consequently, intervention timing may be critical. Given that neurodegenerative processes develop over decades, supplementation initiated during advanced cognitive decline may occur too late to substantially alter disease trajectories, whereas earlier interventions during preclinical or prodromal stages may offer greater potential benefit.

Recent systematic reviews and meta-analyses generally report small-to-moderate but overall favorable effects of omega-3 supplementation, particularly among individuals with MCI or early cognitive decline [173,199]. Furthermore, a recent dose–response meta-analysis of 58 randomized controlled trials suggested that cognitive benefits may be most pronounced at daily doses between 1000 and 2500 mg of DHA and EPA, although substantial heterogeneity and low-to-moderate certainty of evidence limited definitive conclusions [210]. Indeed, significant between-study heterogeneity remains a major limitation across the literature, and evidence for universal cognitive benefits in cognitively healthy older adults is lacking. Overall, current clinical evidence supports a potentially beneficial role for omega-3 fatty acids in cognitive aging, but the magnitude of effect appears highly dependent on baseline nutritional status, stage of cognitive impairment, genetic background, and intervention timing. Future trials incorporating biomarkers of omega-3 status, biological aging, inflammation, and vascular dysfunction may help identify responder populations and facilitate a more precision-based approach to nutritional strategies aimed at promoting cognitive longevity (Table 2 and Table 3).

### 5.3. Why Are Clinical Results Heterogeneous?

Despite strong mechanistic rationale, clinical trials of omega-3 supplementation have produced inconsistent findings. This heterogeneity likely reflects a combination of biological and methodological factors.

Intervention protocols differ substantially with respect to DHA and EPA dosage, formulation, EPA ratio, and treatment duration, making direct comparisons challenging. A recent dose–response meta-analysis suggested that cognitive benefits may be most evident at daily doses between approximately 1000 and 2500 mg, although uncertainty remains regarding optimal dosing strategies and formulations [211,212].

Participant characteristics also appear to influence responsiveness. More favorable outcomes have generally been reported in individuals with mild cognitive impairment, low baseline omega-3 status, or specific genetic backgrounds such as APOE ε4 carriers [196,200,201,211,213]. In addition, substantial interindividual variability exists in omega-3 absorption, metabolism, tissue incorporation, inflammatory burden, vascular health, and biological aging processes, all of which may affect treatment efficacy [89,201,213,214]. The major sources of heterogeneity identified across omega-3 cognitive trials, including intervention timing, baseline omega-3 status, APOE genotype, dose and formulation, intervention duration, outcome selection, biological age, and ceiling effects, are summarized in Table 4.

Overall, inconsistent clinical findings should not necessarily be interpreted as evidence of ineffectiveness. Rather, they likely reflect substantial heterogeneity in study populations, intervention characteristics, and outcome measures. Future studies adopting biomarker-guided and precision nutrition approaches may help identify responder populations and clarify the circumstances under which omega-3 fatty acids are most effective for supporting cognitive health during aging [201,213].

## 6. Omega-3 Fatty Acids Through a Geroscience Lens

### 6.1. Linking Omega-3 Fatty Acids to Hallmarks of Aging

The geroscience hypothesis proposes that interventions targeting fundamental biological mechanisms of aging may simultaneously delay or attenuate multiple age-related diseases and functional declines. Within this framework, omega-3 fatty acids represent promising nutritional modulators of several hallmarks of aging that are closely linked to cognitive decline and neurodegeneration [33,215,216,217].

One of the most extensively studied mechanisms is inflammaging, the chronic low-grade inflammatory state that develops with advancing age. Elevated circulating concentrations of pro-inflammatory cytokines, including interleukin-6 (IL-6), tumor necrosis factor-α (TNF-α), and C-reactive protein (CRP), have been associated with accelerated cognitive decline and increased dementia risk [89,218]. Omega-3 fatty acids can reduce the production of pro-inflammatory mediators and serve as precursors for specialized pro-resolving mediators, including resolvins, protectins, and maresins, thereby promoting the resolution of inflammation and potentially mitigating age-related neuroinflammatory processes [43,44,81].

Omega-3 fatty acids may also influence mitochondrial dysfunction, a key hallmark of aging linked to impaired neuronal energy metabolism and oxidative stress. Experimental evidence suggests that DHA supports mitochondrial membrane integrity, bioenergetic efficiency, and cellular resilience during aging.

Additional evidence suggests interactions with cellular senescence and vascular aging. Omega-3-derived mediators may attenuate senescence-associated inflammatory signaling, while DHA and EPA have been shown to improve endothelial function, reduce vascular inflammation, and support cerebrovascular health, all of which are closely linked to cognitive aging. Emerging research further indicates potential interactions with neural stem cell maintenance and nutrient-sensing pathways, including AMPK, mTOR, and insulin signaling, although direct evidence in human brain aging remains limited [44,75,76,219,220,221]. The potential interactions between omega-3 fatty acids and major hallmarks of aging relevant to cognitive aging and brain health are summarized in Table 5.

**Figure 2:**
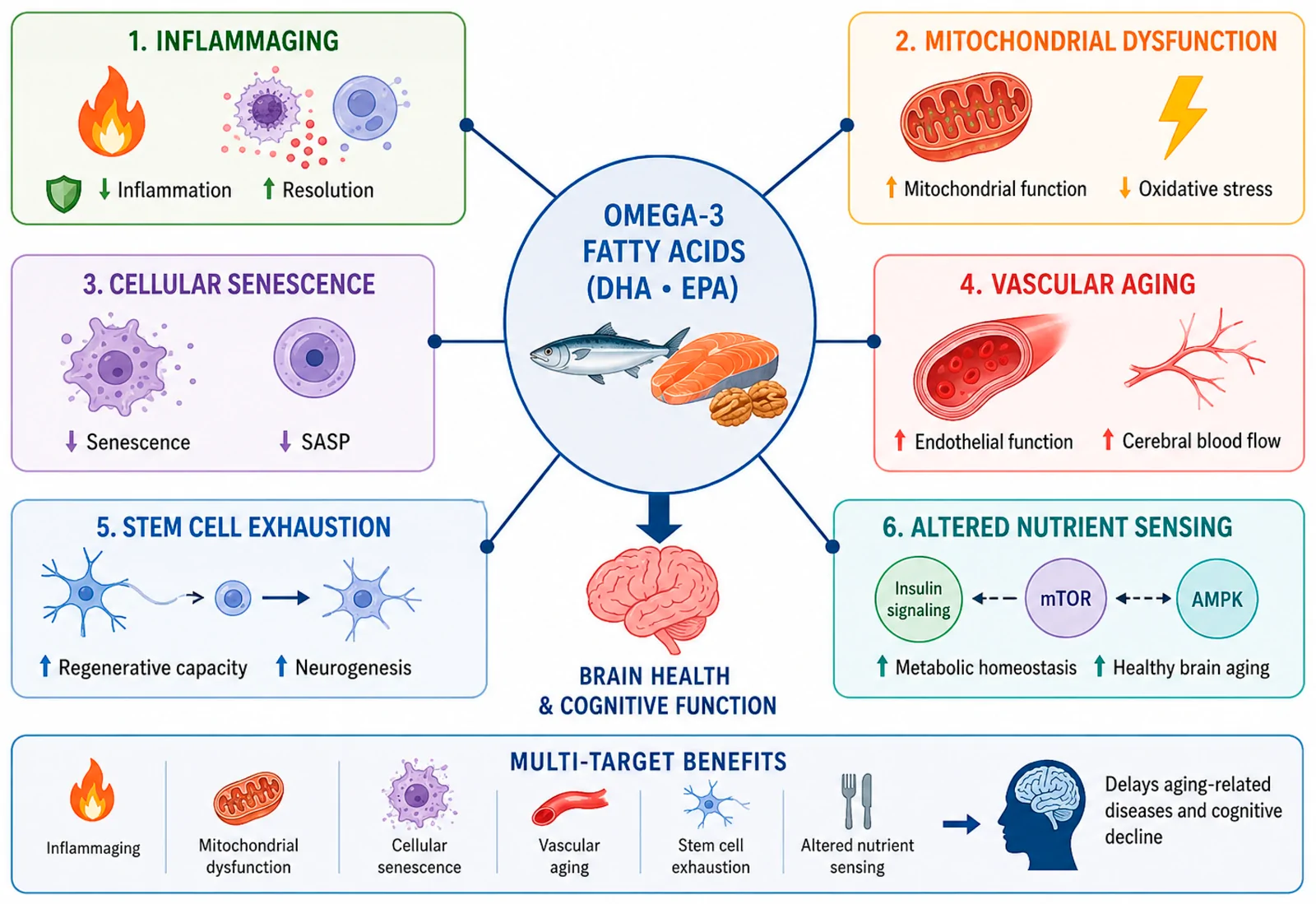
Omega-3 fatty acids as modulators of multiple hallmarks of aging involved in brain health and cognitive function. The figure illustrates the mechanisms through which omega-3 fatty acids modulate molecular hallmarks of brain aging. Omega-3 fatty acids exert pleiotropic effects across multiple biological pathways involved in brain aging. They attenuate chronic low-grade inflammation, improve mitochondrial function, reduce cellular senescence, preserve endothelial function and cerebral perfusion, support neural stem cell maintenance and neurogenesis, and regulate nutrient-sensing pathways, including AMPK, mTOR, and insulin signaling. Collectively, these mechanisms contribute to the maintenance of brain homeostasis, preservation of cognitive function, and attenuation of age-related neurodegenerative processes. Abbreviations: AMPK, AMP-activated protein kinase; DHA, docosahexaenoic acid; EPA, eicosapentaenoic acid; mTOR, mechanistic target of rapamycin; SASP, senescence-associated secretory phenotype. Arrow notation: Upward arrows indicate an increase or enhancement, whereas downward arrows indicate a decrease or attenuation. The panel colors are used only to visually distinguish the different hallmarks of aging.

Collectively, these observations support a geroscience-based model in which omega-3 fatty acids simultaneously influence multiple hallmarks of aging. Through their effects on inflammaging, mitochondrial function, cellular senescence, vascular aging, stem cell maintenance, and nutrient-sensing pathways, omega-3 fatty acids may contribute to the preservation of brain health and cognitive function across the lifespan (Figure 2). Several of these effects are mediated, at least in part, by specialized pro-resolving mediators derived from DHA and EPA, including resolvins, protectins, and maresins; representative chemical structures of these mediators are provided in the Supplementary Materials.

### 6.2. Cognitive Resilience and Healthy Longevity

Cognitive resilience refers to the ability to maintain cognitive function despite age-related neural changes, neuropathology, or other biological stressors affecting the brain. Contemporary frameworks view resilience as a multidimensional construct shaped by genetic, biological, environmental, and lifestyle factors across the lifespan [222,223]. This concept has gained importance because substantial interindividual differences in cognitive outcomes are observed among older adults with similar levels of neuropathology [222,224].

Healthy cognitive aging represents a key component of healthy longevity and is characterized by the maintenance of cognitive abilities, functional independence, and quality of life throughout older adulthood [225]. Although age-related declines in certain cognitive domains are common, cognitive trajectories vary considerably between individuals, indicating that aging-related cognitive changes are not uniform or inevitable [225,226]. Accumulating evidence suggests that modifiable lifestyle factors—including regular physical activity, cognitive stimulation, social engagement, adequate sleep, and adherence to healthy dietary patterns—contribute to the preservation of cognitive function and may delay the onset of cognitive impairment and dementia [15,226].

A central mechanism underlying cognitive resilience is cognitive reserve, defined as the brain’s capacity to optimize or flexibly recruit neural networks in response to aging or neuropathology [222]. Cognitive reserve is influenced by educational attainment, occupational complexity, lifelong learning, cognitively stimulating activities, and social engagement [227,228]. Individuals with greater cognitive reserve generally demonstrate better cognitive performance and increased resilience to age-related cognitive decline and dementia-related pathology [227,228]. Cognitive reserve also appears to interact with other protective factors, including sleep quality, physical activity, and metabolic health, highlighting its role as a potentially modifiable determinant of cognitive healthspan [223,229]. Together, these concepts support a life-course approach to healthy longevity in which maintaining cognitive resilience and cognitive reserve may help preserve functional independence and quality of life during aging [15,227].

### 6.3. Precision Geronutrition

#### 6.3.1. Concept and Rationale of Precision Geronutrition

Precision geronutrition represents the convergence of nutritional science, geroscience, and precision medicine and aims to develop individualized dietary strategies that promote healthy aging and preserve cognitive function [230,231,232]. Unlike conventional population-based recommendations, precision approaches consider interindividual differences in genetics, metabolic status, lifestyle, and age-related physiological changes to optimize nutritional interventions [230,233]. Such strategies seek to tailor dietary interventions according to biological characteristics rather than chronological age and are increasingly regarded as an important component of healthy longevity research. The implementation of precision geronutrition is facilitated by advances in systems biology, high-throughput omics technologies, and computational approaches that enable the integration of multidimensional health data [231].

#### 6.3.2. Biomarkers and Biological Age in Nutritional Geroscience

The identification of biomarkers of aging is a central component of precision geronutrition because aging involves multiple interconnected biological pathways rather than a single mechanism [234,235]. No single biomarker has proven sufficient to comprehensively characterize healthy aging, highlighting the need for multidimensional approaches that integrate functional, biochemical, and molecular indicators [234]. Composite biomarker panels are increasingly preferred over individual markers for evaluating aging trajectories and responses to interventions [234,235].

Biological age often predicts functional decline, morbidity, and mortality more accurately than chronological age [236]. Epigenetic clocks, proteomic signatures, metabolomic profiles, and functional biomarkers have emerged as promising indicators of biological aging [235,236].

Because nutrition influences oxidative stress, inflammation, mitochondrial function, and epigenetic regulation, personalized dietary interventions may contribute to slowing biological aging and extending healthspan [235,236].

#### 6.3.3. Genetic Determinants of Nutritional Responses: The Role of APOE

Genetic variability contributes substantially to heterogeneity in nutritional responses and susceptibility to age-related diseases [237]. Nutrigenomic studies indicate that genetic differences influence nutrient metabolism, inflammatory pathways, and disease risk [237].

Among genetic factors, apolipoprotein E (APOE) is particularly important in cognitive aging and Alzheimer’s disease [238,239]. The APOE ε4 allele represents the strongest common genetic risk factor for late-onset Alzheimer’s disease [238]. APOE genotype affects lipid metabolism, neuroinflammation, and amyloid accumulation and may modulate responses to dietary interventions [238,239,240]. Although personalized nutritional strategies based on APOE status are being investigated, current evidence remains insufficient to support genotype-specific dietary recommendations in clinical practice [238,239,241].

#### 6.3.4. Inflammaging Phenotypes and Personalized Dietary Interventions

Inflammaging refers to the chronic low-grade inflammatory state that accompanies aging and contributes to the development of age-related diseases [83,89,242]. Multiple mechanisms, including cellular senescence, mitochondrial dysfunction, microbiome alterations, immune dysregulation, and oxidative stress, contribute to the development of inflammaging [83,89,243,244].

Considerable interindividual variability exists in inflammatory trajectories, giving rise to distinct inflammaging phenotypes that are shaped by genetics, lifestyle, environmental exposures, and lifelong immunobiography [83,242,245]. Individuals exhibiting pro-inflammatory phenotypes are particularly susceptible to frailty, cardiovascular disease, metabolic disorders, multimorbidity, and cognitive decline [89,243,244]. These observations support the implementation of precision nutrition approaches aimed at modulating chronic inflammation and promoting healthy aging [83,242,246].

Dietary patterns rich in polyphenols, dietary fiber, omega-3 fatty acids, and plant-derived foods may exert anti-inflammatory effects and may be especially beneficial in individuals with elevated inflammatory burden [242,246,247]. Emerging inflammatory clocks and biomarker-guided strategies may facilitate the identification of responders and non-responders to personalized dietary interventions [242,245,248].

#### 6.3.5. Multi-Omics Approaches in Precision Geronutrition

Recent advances in omics technologies have accelerated the development of precision geronutrition [231,249,250,251]. Genomics, epigenomics, transcriptomics, proteomics, metabolomics, and microbiome analyses provide complementary information regarding the biological mechanisms underlying aging and cognitive resilience [249,250,251,252].

The integration of multi-omics datasets may enable the identification of molecular signatures associated with healthy aging and differential responses to dietary interventions [250,251,252,253]. Systems biology approaches are expected to facilitate the transition from generalized dietary recommendations toward individualized nutritional strategies aimed at extending healthspan and preserving cognitive function [231,251,253]. However, the clinical implementation of multi-omics approaches remains limited by methodological heterogeneity, high cost, lack of standardization, and the need for longitudinal validation studies [249,250,252].

#### 6.3.6. Towards Precision Geronutrition

Emerging evidence suggests that the benefits of omega-3 fatty acids may not be uniform across populations and that certain biological and clinical phenotypes may exhibit greater responsiveness to nutritional interventions [201,211,213]. Individuals with low baseline omega-3 status, particularly those with a low omega-3 index, may derive greater benefit from supplementation than individuals with adequate tissue levels, supporting the baseline deficiency hypothesis [202,204,213,254].

APOE ε4 carriers represent another potential responder subgroup because APOE genotype influences lipid transport, neuroinflammation, and brain DHA metabolism, although evidence remains heterogeneous [196,238,239]. Individuals with mild cognitive impairment (MCI) may represent a particularly promising target population, as interventions initiated during prodromal stages may be more effective than those introduced after established dementia [199,200,201].

Older adults exhibiting pro-inflammatory or inflammaging phenotypes may also be more responsive to omega-3 interventions because DHA and EPA exert anti-inflammatory and pro-resolving actions [83,242]. Individuals with vascular dysfunction or increased cerebrovascular risk may derive additional benefit from omega-3 fatty acids owing to their favorable effects on endothelial function and vascular homeostasis [137,138,255].

Metabolic disturbances, including insulin resistance, may further modify responses to omega-3 interventions because impaired metabolic homeostasis contributes to inflammation and neurodegeneration [44,219,231].

Finally, biological age acceleration may provide a more informative framework than chronological age for identifying individuals who could benefit most from interventions targeting multiple hallmarks of aging simultaneously [33,234,235,236]. Taken together, these observations support a precision geronutrition framework in which omega-3 supplementation may be most effective in biologically vulnerable individuals rather than in unselected populations, although further biomarker-guided clinical trials are needed to validate these responder phenotypes [201,213,235].

## 7. Clinical and Public Health Implications

Current evidence supports the importance of nutritional strategies for healthy cognitive aging; however, the available data remain insufficient to recommend universal omega-3 supplementation for the prevention of cognitive decline or dementia [211,213]. Although epidemiological studies generally suggest favorable associations, randomized controlled trials have yielded heterogeneous findings, emphasizing the need for cautious interpretation [173,256]. Therefore, a food-first approach integrated within overall healthy dietary patterns currently represents the most evidence-supported strategy [213,257].

### 7.1. Dietary Recommendations and Mediterranean-Type Dietary Patterns

Accumulating evidence supports Mediterranean-style dietary patterns as a cornerstone of healthy brain aging [182,258]. These dietary patterns emphasize regular consumption of fish and seafood, vegetables, fruits, legumes, whole grains, nuts, and olive oil, while limiting ultra-processed foods and saturated fats [182,259]. The cognitive benefits associated with Mediterranean dietary patterns are likely attributable to synergistic interactions among multiple nutrients and bioactive compounds, including omega-3 fatty acids, polyphenols, vitamins, and antioxidants rather than to individual nutrients alone [182,258].

From a public health perspective, recommendations should focus on improving overall dietary quality instead of promoting isolated nutrients [182,257]. Such dietary approaches are consistent with contemporary concepts of precision nutrition and healthy aging promoted by geroscience frameworks [260].

### 7.2. Fish Consumption and Food-First Approaches

Regular fish consumption remains the preferred strategy for achieving adequate intake of DHA and EPA [44,257]. Fatty fish such as salmon, sardines, herring, and mackerel provide not only long-chain omega-3 fatty acids but also vitamin D, selenium, iodine, and high-quality protein, all of which may contribute to healthy aging and brain function [193,257]. Several international dietary guidelines recommend consuming one to two servings of fish per week, with an emphasis on oily fish species [257,261].

Importantly, observational studies have generally demonstrated stronger and more consistent associations between fish consumption and cognitive outcomes than supplementation trials, suggesting that whole-food matrices and broader dietary patterns may substantially contribute to the observed benefits [173,175]. This observation highlights the importance of dietary context and potential synergistic interactions among nutrients [182,258].

### 7.3. Supplementation Considerations

Despite extensive interest in omega-3 supplements, current evidence does not support routine supplementation for all older adults [211,256]. Recent systematic reviews indicate that supplementation may provide modest benefits in selected individuals, particularly those with low baseline omega-3 status, mild cognitive impairment, or increased cardiometabolic risk, whereas consistent cognitive benefits have not been demonstrated in cognitively healthy populations [211,256]. Moreover, considerable heterogeneity exists among clinical trials regarding dosage, duration, formulation, baseline nutritional status, and outcome measures, which may partly explain inconsistent findings [173].

Consequently, individualized strategies considering dietary habits, nutritional status, comorbidities, genetic susceptibility, and patient preferences appear more appropriate than universal recommendations [33,173]. Future precision nutrition approaches may help identify subgroups most likely to benefit from omega-3 interventions [33,260].

### 7.4. Safety Considerations

Omega-3 fatty acids are generally considered safe and well tolerated when consumed through foods or within recommended supplemental doses [256]. Mild gastrointestinal symptoms represent the most commonly reported adverse effects [256]. High-dose supplementation may increase bleeding risk in susceptible individuals and should therefore be used cautiously in patients receiving anticoagulant or antiplatelet therapy [262]. Furthermore, recent cardiovascular studies have raised concerns regarding potential associations between very high doses of omega-3 fatty acids and atrial fibrillation, underscoring the importance of individualized risk–benefit assessment [262,263].

### 7.5. Sustainability and Healthy Aging

Sustainability has emerged as an increasingly important consideration in nutritional recommendations [264]. Concerns regarding overfishing, environmental degradation, and marine ecosystem preservation have stimulated interest in alternative omega-3 sources, including algae-derived DHA and EPA, which may represent environmentally sustainable options for individuals with low fish intake or plant-based dietary preferences [55,264]. Sustainable dietary patterns, including Mediterranean-type diets, may simultaneously support both planetary and brain health [264].

Because older adults are particularly vulnerable to malnutrition and inadequate nutrient intake, maintaining sufficient intake of protein, micronutrients, and omega-3 fatty acids should be considered an integral component of healthy aging strategies [265]. Importantly, dietary interventions should be individualized and integrated with other lifestyle factors, including physical activity, vascular risk management, sleep quality, and social engagement, all of which interact to influence cognitive trajectories during aging [33,265].

Overall, current evidence favors a conservative food-first approach emphasizing Mediterranean-type dietary patterns and regular fish consumption rather than routine supplementation [211,213]. Although omega-3 supplementation may be appropriate in selected individuals, the available evidence remains insufficient to support universal supplementation for the prevention of cognitive decline or dementia [211,256].

## 8. Limitations of Current Evidence

Omega-3 polyunsaturated fatty acids, particularly eicosapentaenoic acid and docosahexaenoic acid, have been extensively investigated as potential nutritional interventions for cognitive aging, neuroprotection, and dementia prevention [174,211,266]. Despite strong biological plausibility and generally favorable findings from observational studies, the current evidence base remains limited by several methodological, biological, and translational challenges that complicate interpretation of clinical outcomes [174,211,266,267,268].

A major limitation is the substantial heterogeneity among randomized controlled trials. Study populations differ markedly with respect to age, baseline cognitive status, vascular risk profile, APOE genotype, comorbidities, and habitual dietary intake, all of which may influence responsiveness to omega-3 supplementation [174,211,266]. Importantly, individuals with mild cognitive impairment, low baseline omega-3 status, or increased biological vulnerability may respond differently from cognitively healthy and well-nourished older adults, yet many trials have not been adequately designed or powered to identify these responder populations [201,211]. Consequently, substantial between-study heterogeneity reduces comparability and reproducibility of findings and remains one of the principal challenges in interpreting the literature [174,211,267].

Another important limitation relates to intervention characteristics. Considerable variation exists in DHA and EPA dosage, EPA:DHA ratio, formulation, treatment duration, and adherence assessment across studies [174,211]. Furthermore, many interventions have lasted only 6–24 months, whereas neurodegenerative processes and cognitive decline evolve over decades [266,267]. As a result, existing studies may underestimate the long-term effects of omega-3 fatty acids on brain aging trajectories and dementia risk [174,268].

Methodological heterogeneity further complicates interpretation of findings. Cognitive aging affects multiple domains, including episodic memory, executive function, attention, and processing speed, yet studies employ highly variable neuropsychological batteries and outcome definitions [174]. This lack of standardization limits direct comparisons across trials and may reduce sensitivity for detecting subtle or domain-specific cognitive effects. In addition, ceiling effects may obscure potential benefits in cognitively healthy populations where baseline performance is already high [211].

Baseline omega-3 status represents a particularly important but frequently overlooked source of bias. Many studies do not measure or stratify participants according to circulating omega-3 concentrations prior to intervention [174,268]. Consequently, supplementation effects may be diluted when individuals with adequate omega-3 status are included alongside potentially deficient participants who may derive greater benefit from supplementation [174,268]. Future studies should therefore incorporate objective biomarkers, such as the omega-3 index, to improve participant characterization and stratification [211,268].

Biomarker limitations represent another important gap. Most clinical trials rely primarily on cognitive endpoints while providing limited information regarding biological pathways potentially influenced by omega-3 fatty acids, including neuroinflammation, endothelial dysfunction, oxidative stress, lipid mediator production, and neurodegenerative processes [44,219,268]. The absence of biomarker-guided analyses makes it difficult to determine whether neutral cognitive findings reflect biological inefficacy or insufficient study sensitivity to detect mechanistic changes [44,268].

A further challenge relates to translational limitations. Much of the mechanistic evidence supporting omega-3 fatty acids originates from cellular and animal studies, where anti-inflammatory, neuroprotective, and vasoprotective effects are consistently observed [44,219]. However, translation of these findings into clinically meaningful cognitive benefits in humans has been less consistent [173,211]. This discrepancy highlights the complexity of human cognitive aging and suggests that favorable mechanistic effects do not necessarily translate into measurable short-term cognitive improvements, particularly in heterogeneous aging populations [173,211].

Importantly, most existing studies are not embedded within a geroscience framework and therefore evaluate disease-specific outcomes rather than biological aging processes such as inflammaging, cellular senescence, mitochondrial dysfunction, or metabolic dysregulation. As a result, potentially important effects of omega-3 fatty acids on fundamental aging mechanisms may remain undetected when only conventional cognitive endpoints are assessed [33,219].

Finally, publication bias, selective outcome reporting, and the predominance of secondary cognitive analyses may further influence the published literature, particularly in smaller studies or those supported by industry funding. Positive findings may be more likely to be reported than neutral or negative results, potentially contributing to overestimation of treatment efficacy [266,267].

**Figure 3:**
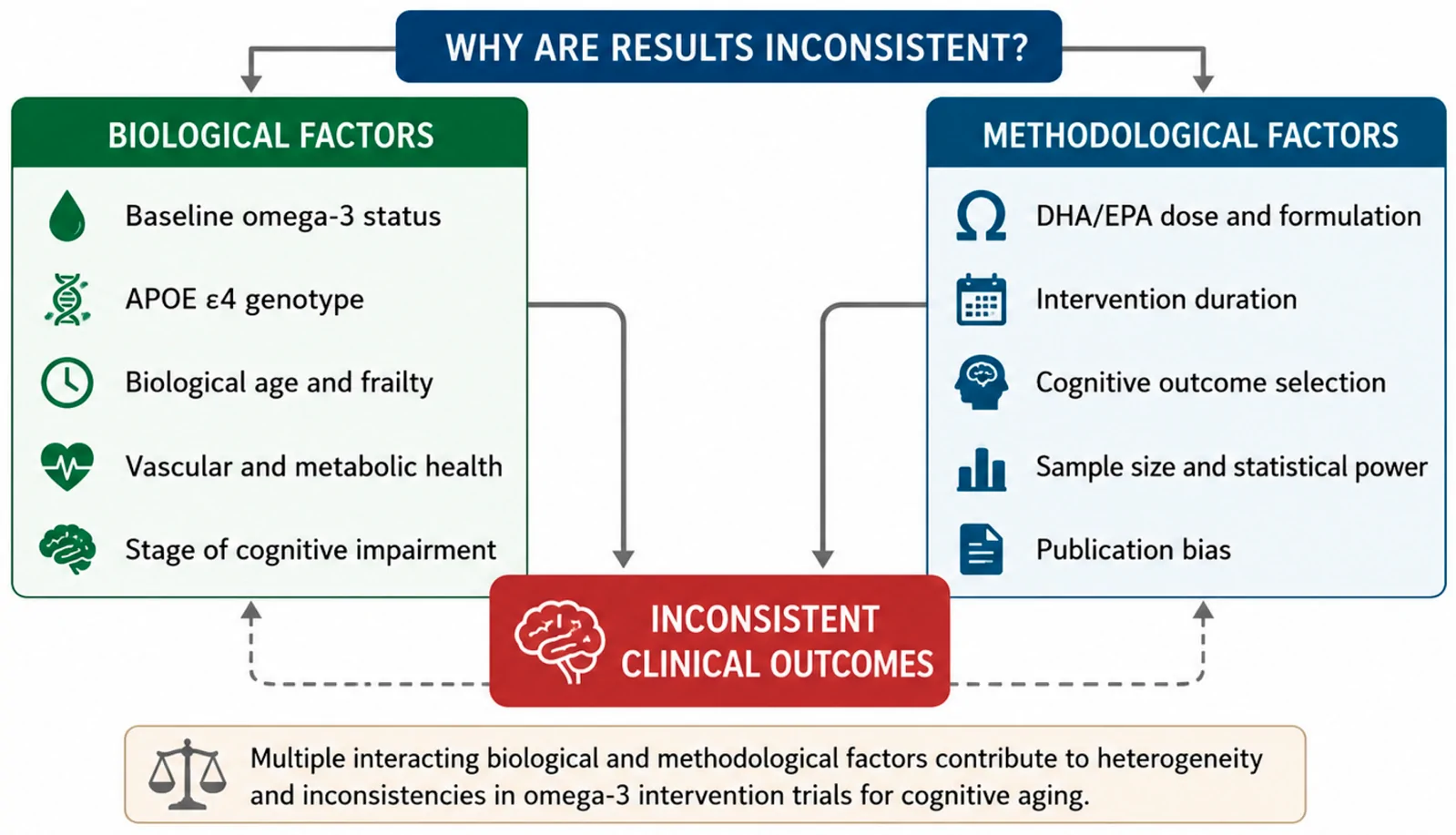
Major sources of uncertainty contributing to inconsistent findings in omega-3 intervention trials targeting cognitive aging. Clinical outcomes are influenced by multiple interacting methodological and biological factors, including population heterogeneity, baseline omega-3 status, APOE genotype, intervention timing, dosage and formulation, treatment duration, adherence, dietary background, biomarker availability, translational differences between experimental and clinical studies, the absence of a geroscience framework, and publication bias. These factors collectively contribute to the variability observed across randomized clinical trials and may partly explain the discrepancy between strong mechanistic evidence and inconsistent cognitive outcomes in humans. Arrow notation: Solid arrows indicate the contribution of biological and methodological factors to inconsistent clinical outcomes, whereas dashed arrows indicate reciprocal interactions and feedback. The colors are used only to visually distinguish biological from methodological factors.

Taken together, these limitations suggest that the absence of consistent clinical benefits should not necessarily be interpreted as evidence of ineffectiveness. Rather, current findings likely reflect substantial biological and methodological heterogeneity across study populations, intervention protocols, and outcome measures [211]. Future research should adopt precision-nutrition approaches incorporating baseline omega-3 status, genetic profiling, biomarkers of biological aging, and longer follow-up periods to better identify populations most likely to benefit from omega-3 interventions [211]. The major methodological and biological factors contributing to the heterogeneous findings observed across omega-3 intervention studies are summarized in Figure 3. These interacting sources of variability should be considered when interpreting the inconsistent effects of omega-3 fatty acids on cognitive aging and dementia prevention.

## 9. Challenges and Future Directions

Future studies should prioritize longitudinal mechanistic investigations integrating nutritional exposures with molecular, metabolic, and functional trajectories to better understand the role of omega-3 fatty acids in cognitive aging and healthy longevity [33,250,269]. Multidomain interventions combining dietary optimization with physical activity, cognitive training, and vascular risk management may provide greater benefits than isolated nutritional approaches and should be further explored in large-scale clinical trials [270,271,272].

The development of biomarker-guided supplementation strategies based on circulating fatty acids, metabolomic profiles, and inflammaging biomarkers may improve the identification of individuals most likely to benefit from intervention [235,248,269,273]. Future geroscience-informed clinical trials should incorporate biological age metrics, including epigenetic and proteomic clocks, as well as neuroimaging biomarkers, to evaluate intervention effects on aging trajectories rather than disease-specific outcomes alone [236,242,246,273]. Advances in precision nutrition and multi-omics technologies are expected to facilitate the transition from generalized recommendations toward individualized dietary strategies aimed at preserving cognitive function and extending healthspan (Table 6) [230,231].

## 10. Conclusions

Omega-3 fatty acids are biologically plausible modulators of several mechanisms implicated in cognitive aging, including chronic inflammation, vascular dysfunction, oxidative stress, mitochondrial dysfunction, and altered intercellular communication [43,44,267]. Experimental and observational studies generally support a potential role for omega-3 fatty acids in maintaining brain health; however, these findings have not been consistently translated into clinically meaningful cognitive benefits in randomized controlled trials [174,211,266]. Current evidence does not support the routine use of omega-3 supplementation as a universal strategy for the prevention of cognitive decline or dementia in older adults [174,211]. Although some studies have reported favorable effects on specific cognitive domains, biological markers, or neurodegeneration-related outcomes, overall trial results remain inconsistent and effect sizes are generally small [173]. The most promising signals have been observed in selected populations, including individuals with mild cognitive impairment, low baseline omega-3 status, APOE ε4 carriers, and those with increased biological vulnerability, suggesting that responsiveness to omega-3 supplementation may not be uniform across aging populations [174,201,211,213].

Taken together, the available evidence suggests that omega-3 fatty acids should currently be viewed as a potentially useful component of precision nutrition approaches rather than a broadly effective intervention for cognitive aging [201,211,213]. Future studies should prioritize biomarker-guided participant selection, assessment of baseline omega-3 status, integration of biological aging metrics, and longer-term multidomain interventions to determine whether specific subgroups derive meaningful cognitive benefit from supplementation [33,230,231,235]. Such approaches may help clarify the role of omega-3 fatty acids in promoting cognitive resilience and healthy longevity while addressing the substantial heterogeneity that currently limits interpretation of the evidence base [173,174,211].

## Figures and Tables

**Table 1:** Key observational studies investigating the relationship between omega-3 fatty acids and cognitive ageing, cognitive decline, and dementia.

Study	Population	Exposure	Outcome	Main Findings	Limitations
Kalmijn et al., 1997 [179]	Rotterdam Study participants	Dietary fish and fatty acid intake	Incident dementia	Higher fish consumption was associated with a lower risk of dementia.	Dietary assessment may be subject to measurement error; residual confounding cannot be excluded.
Morris et al., 2003 [192]	815 older adults (Chicago Health and Aging Project)	Fish consumption and dietary omega-3 intake	Incident Alzheimer’s disease	Regular fish consumption was associated with a reduced risk of Alzheimer’s disease.	Dietary intake was self-reported; observational design precludes causal inference.
Schaefer et al., 2006 [188]	899 dementia-free participants (Framingham Study)	Plasma phosphatidylcholine DHA concentration	Incident dementia and Alzheimer’s disease	Higher plasma DHA levels were associated with a lower risk of dementia and Alzheimer’s disease.	Single baseline biomarker measurement may not reflect long-term exposure.
Barberger-Gateau et al., 2007 [193]	1416 older adults (PAQUID cohort)	Fish consumption	Incident dementia	Regular fish intake was associated with a lower risk of dementia.	Potential dietary misclassification and lifestyle-related confounding.
van Gelder et al., 2007 [180]	210 elderly men (Zutphen Elderly Study)	Fish consumption and dietary EPA+DHA intake	Five-year cognitive decline	Fish consumption and higher EPA+DHA intake were associated with less cognitive decline during follow-up.	Small, male-only cohort; findings may not be generalisable to women or other populations.
Tan et al., 2012 [185]	1575 older adults (Cardiovascular Health Study)	Red blood cell omega-3 fatty acid levels	Brain MRI markers and cognitive function	Higher DHA levels were associated with larger brain volumes and healthier brain ageing.	Brain imaging markers were used rather than incident dementia outcomes.
Samieri et al., 2012 [194]	1214 older adults (Three-City Study)	Plasma long-chain omega-3 fatty acids	Medial temporal lobe atrophy	Higher plasma EPA levels were associated with less gray matter atrophy in the hippocampal–parahippocampal region and amygdala.	Observational design; associations differed according to individual omega-3 fatty acids, and residual confounding cannot be excluded.
Lai et al., 2018 [184]	2622 older adults (Cardiovascular Health Study)	Serial plasma phospholipid omega-3 PUFA concentrations	Healthy ageing	Higher long-term omega-3 status was associated with an increased likelihood of healthy ageing.	Cognitive outcomes were assessed as part of a broader healthy ageing construct.
Thomas et al., 2021 [187]	1279 participants (Three-City Study)	Blood long-chain omega-3 polyunsaturated fatty acids	Brain atrophy, cognitive decline, and incident dementia	Higher plasma EPA+DHA levels were associated with slower global cognitive decline, less medial temporal lobe atrophy, and a lower risk of incident dementia.	Observational design; residual confounding and reverse causation cannot be completely excluded.
Liu et al., 2022 [178]	215,083 older adults (UK Biobank)	Fish oil supplementation	Incident dementia	Regular fish oil supplementation was associated with a lower risk of incident dementia.	Supplement use was self-reported; healthy-user bias remains possible.
He et al., 2023 [109]	440,750 participants (UK Biobank)	Circulating polyunsaturated fatty acids and fish oil supplementation	Incident dementia	Higher omega-3 status was associated with a lower risk of dementia after multivariable adjustment.	Observed effect sizes were modest after adjustment for potential confounders.
Sala-Vila et al., 2023 [189]	267,312 participants (UK Biobank)	Plasma omega-3 fatty acids	Incident dementia	Higher circulating omega-3 levels were associated with a lower risk of all-cause dementia.	Observational design cannot establish causality.

Abbreviations: AD, Alzheimer’s disease; DHA, docosahexaenoic acid; PUFA, polyunsaturated fatty acid. Note: Most observational studies report favorable associations between higher dietary or circulating omega-3 fatty acid levels and cognitive outcomes. However, interpretation should consider residual confounding, healthy-user bias, reverse causation, and overlap with broader healthy dietary patterns.

**Table 2:** Major randomized controlled trials investigating the effects of omega-3 supplementation on cognition and brain function across adulthood and aging.

Trial	Sample Size	Population	Intervention	Duration	Cognitive Outcomes	Main Conclusions
Arellanes et al., 2020 (EBioMedicine) [195]	n = 33	Cognitively unimpaired adults aged ≥55 years with a first-degree family history of dementia and additional dementia risk factors	DHA 2152 mg/day plus vitamin B complex vs. placebo plus vitamin B complex	6 months	Cognitive performance; CSF DHA concentrations	DHA supplementation increased CSF DHA and EPA levels, but did not significantly improve cognition or brain structural measures.
Mengelberg et al., 2022 (Int J Geriatr Psychiatry) [196]	n = 72	Older adults with mild cognitive impairment (MCI)	DHA 1491 mg/day + EPA 351 mg/day vs. placebo	12 months	Global cognition, mood, well-being	No significant improvement in global cognition; favorable effects on depressive symptoms, anxiety, and vascular parameters in some subgroups.
Lin et al., 2022 (Brain Behav Immun) [197]	n = 57	Patients with MCI or early Alzheimer’s disease	DHA, EPA, DHA + EPA, or placebo	6 months	Cognitive function and blood-based biomarkers	Cognitive effects were limited, although favorable changes were observed in selected blood-based biomarkers.
Power et al., 2022 (Clin Nutr) [205]	n = 60	Cognitively healthy older adults	Omega-3 fatty acids + carotenoids + vitamin E vs. placebo	24 months	Working memory, executive function	Improvements in working memory were observed; contribution of omega-3 alone could not be isolated.
Vauzour et al., 2023 (Am. J. Clin. Nutr.) [206]	n = 259	Older adults with subjective memory complaints	DHA-rich fish oil, cocoa flavanols, their combination, or placebo	12 months	Global cognition, memory, brain imaging outcomes	Neither intervention alone nor in combination significantly improved cognitive performance or brain structural measures.
Shinto et al., 2024 (JAMA Netw Open) [201]	n = 102	Older adults with low omega-3 status and cerebral white matter lesions	975 mg EPA + 650 mg DHA/day vs. placebo	3 years	White matter lesion progression, neuronal integrity biomarkers	No significant overall effect was observed; subgroup analysis suggested less neuronal integrity decline among APOE ε4 carriers.
Duan et al., 2025 (J. Affect. Disord.) [200]	n = 280	Older adults with MCI	DHA 800 mg/day, medium-chain triglycerides, combination therapy, or placebo	12 months	Global cognition, memory, executive function	Combination of DHA and medium-chain triglycerides produced greater cognitive benefits than either intervention alone.
Danthiir et al., 2018 (Am J Clin Nutr) [209]	n = 390	Cognitively healthy adults aged 65–90 years	DHA-rich fish oil (1720 mg DHA + 600 mg EPA/day) vs. placebo	18 months	Memory, executive function, processing speed	No significant effects on age-related cognitive decline despite substantial increases in omega-3 status.
van de Rest et al., 2008 (Neurology) [208]	n = 302	Cognitively healthy older adults	Fish oil providing approximately 400 or 1800 mg EPA + DHA/day vs. placebo	26 weeks	Memory, attention, executive function	Neither dose of EPA+DHA significantly improved cognitive performance compared with placebo.
Boespflug et al., 2016 (J Nutr Health Aging) [101]	n = 21	Older adults with subjective memory impairment	Fish oil providing 1.4 g EPA and 1.0 g DHA/day vs. placebo	24 weeks	Working memory performance; fMRI activation	Fish oil supplementation improved working memory performance and increased posterior cingulate cortex activation during memory tasks.
MAPT Trial Andrieu et al., 2017 [207]	n = 1680	Community-dwelling adults aged ≥70 years with memory complaints or other indicators of increased cognitive risk	Omega-3 supplementation, multidomain intervention, both interventions, or placebo	3 years	Composite cognitive decline, memory, executive function, and global cognition	Omega-3 supplementation and the multidomain intervention, alone or combined, did not significantly reduce cognitive decline in the overall population.
Stonehouse et al., 2013 * [202]	n = 176	Healthy adults aged 18–45 years with low habitual DHA intake	DHA 1.16 g/day vs. placebo	6 months	Episodic and working memory, memory reaction time	DHA improved episodic-memory reaction time; memory accuracy improved in women and working-memory reaction time improved in men.

* This study included younger adults and was retained to illustrate potential age- and sex-dependent cognitive responses to DHA supplementation. Abbreviations: DHA, docosahexaenoic acid; EPA, eicosapentaenoic acid; MCI, mild cognitive impairment; PUFA, polyunsaturated fatty acids; CSF, cerebrospinal fluid; APOE, apolipoprotein E; fMRI, functional magnetic resonance imaging.

**Table 3:** Key Messages from Randomized Controlled Trials.

Key Finding	Interpretation
Benefits are most consistently observed in individuals with mild cognitive impairment	Early cognitive decline may represent a therapeutic window for intervention
Low baseline omega-3 status may predict responsiveness	Targeted approaches may be more effective than universal supplementation
APOE ε4 carriers may exhibit differential responses	Genetic factors may influence treatment efficacy
Earlier intervention appears more favorable than late-stage treatment	Preventive strategies may offer greater benefits than interventions initiated after established dementia

Abbreviation: APOE ε4, apolipoprotein E ε4 allele.

**Table 4:** Major sources of heterogeneity in omega-3 cognitive trials.

Factor	Potential Impact
Intervention timing	Earlier may be better
Baseline omega-3 status	Deficient individuals may benefit more
APOE genotype	Differential response
Dose and formulation	Variable efficacy
Intervention duration	Longer follow-up needed
Outcome measures	Reduced comparability
Biological age	Different responsiveness
Ceiling effects	Reduced detectability

Abbreviation: APOE, apolipoprotein E.

**Table 5:** Potential interactions between omega-3 fatty acids and major hallmarks of aging relevant to cognitive aging and brain health.

Hallmark of Aging	Potential Omega-3 Effects	Evidence Level	Human Evidence	Relevance to Cognitive Aging
Chronic inflammation (inflammaging)	Reduced IL-6, TNF-α and CRP; generation of specialized pro-resolving mediators (resolvins, protectins, maresins)	Strong	Moderate–strong	Neuroinflammation, cognitive decline
Mitochondrial dysfunction	Improved membrane composition, oxidative phosphorylation, reduced ROS production	Moderate	Limited	Neuronal energy metabolism and resilience
Cellular senescence	Attenuation of SASP-associated inflammatory signaling and oxidative stress	Moderate	Limited	Chronic neuroinflammation and tissue dysfunction
Altered intercellular communication	Modulation of immune signaling, microglial activation and lipid mediators	Strong	Moderate	Synaptic dysfunction and inflammaging
Stem cell exhaustion	Support of neurogenesis and neural stem cell survival in experimental models	Limited	Absent	Reduced regenerative capacity
Deregulated nutrient sensing	Interactions with AMPK, mTOR and insulin signaling pathways	Moderate	Limited	Metabolic dysfunction and brain aging
Loss of proteostasis	Indirect reduction in oxidative stress and support of mitochondrial quality control	Limited	Absent	Protein aggregation and neurodegeneration
Disabled macroautophagy	Possible enhancement of autophagy and mitophagy pathways in experimental studies	Limited	Absent	Accumulation of damaged organelles
Dysbiosis and gut–brain axis alterations *	Increased microbial diversity and anti-inflammatory metabolites	Moderate	Emerging	Neuroimmune communication
Vascular aging and endothelial dysfunction *	Improved endothelial function, nitric oxide bioavailability and cerebral perfusion	Strong	Moderate	Vascular cognitive impairment

* Although not included among the original hallmarks of aging, dysbiosis and vascular aging are increasingly recognized as important mechanisms contributing to age-related cognitive decline and are highly relevant within contemporary geroscience frameworks. Abbreviations: AMPK, AMP-activated protein kinase; CRP, C-reactive protein; IL-6, interleukin-6; mTOR, mechanistic target of rapamycin; ROS, reactive oxygen species; SASP, senescence-associated secretory phenotype; TNF-α, tumor necrosis factor-alpha.

**Table 6:** Key clinical implications of food-derived omega-3 fatty acids for cognitive aging.

Clinical Implication	Practical Message
Prefer dietary sources	Regular consumption of fatty fish and other omega-3-rich foods remains the preferred strategy for maintaining adequate omega-3 status.
Identify individuals with low omega-3 status	Older adults with low dietary intake or low circulating omega-3 levels may derive the greatest benefit from increasing omega-3 exposure.
Focus on early stages	Evidence appears strongest in individuals with mild cognitive impairment or early cognitive decline rather than established dementia.
Consider precision approaches	Baseline nutritional status, APOE genotype, vascular health, and biological age may influence responsiveness to omega-3 interventions.
Use within multidomain prevention	Omega-3 intake should be considered one component of a broader healthy-aging strategy that includes physical activity, vascular risk-factor control, adequate sleep, and cognitive and social engagement.

Abbreviation: APOE, apolipoprotein E.

## Data Availability

No new data were created or analyzed in this study. Data sharing is not applicable to this article.

## Supplementary Materials

The following supporting information can be downloaded at: https://www.mdpi.com/article/10.3390/nu18162594/s1, Figure S1: Representative chemical structures of omega-3 fatty acid-derived specialized pro-resolving mediators: (A) resolvin D1 (RvD1), (B) protectin D1/neuroprotectin D1 (PD1/NPD1), and (C) maresin 1 (MaR1). The chemical structure images are reproduced with permission from Cayman Chemical Company, Inc. [274,275,276].

## Abbreviations

AD	Alzheimer’s Disease
APOE	Apolipoprotein E
ARA	Arachidonic Acid
BBB	Blood–Brain Barrier
BDNF	Brain-Derived Neurotrophic Factor
CNS	Central Nervous System
DHA	Docosahexaenoic Acid
EPa	Eicosapentaenoic Acid
FADS	Fatty Acid Desaturase
IL-6	Interleukin-6
LC-PUFAs	Long-Chain Polyunsaturated Fatty Acids
LCPUFAs	Long-Chain Polyunsaturated Fatty Acids
MCI	Mild Cognitive Impairment
MRI	Magnetic Resonance Imaging
NF-κB	Nuclear Factor Kappa B
NIA-AA	National Institute on Aging–Alzheimer’s Association
OM3	Omega-3 Fatty Acids
PUFAs	Polyunsaturated Fatty Acids
RCT	Randomized Controlled Trial
ROS	Reactive Oxygen Species
SPMs	Specialized Pro-Resolving Mediators
TNF-α	Tumor Necrosis Factor Alpha
WHO	World Health Organization
DNA	Deoxyribonucleic Acid
RNA	Ribonucleic Acid
mTOR	Mechanistic Target of Rapamycin
AMPK	AMP-Activated Protein Kinase
NAD+	Nicotinamide Adenine Dinucleotide
SASP	Senescence-Associated Secretory Phenotype
OMICS	Multi-omics Approaches
GWAS	Genome-Wide Association Study
