# Supplementary material for: Food-Derived Omega-3 Fatty Acids and Cognitive Aging: Integrating Nutritional Neuroscience and Geroscience
Source: Nutrients. 2026 Aug 7;18(16):2594. doi: 10.3390/nu18162594 (PMC13516186; doi:10.3390/nu18162594)
Supplement: Supplementary file 1 [file nutrients-18-02594-s001.zip › nutrients-4425469-supplementary.pdf]

Figure S1. Representative chemical structures of omega-3 fatty acid-derived specialized pro-resolving mediators: (A) resolvin D1 (RvD1), (B) protectin D1/neuroprotectin D1 (PD1/NPD1), and (C) maresin 1 (MaR1). Permission to reproduce the chemical structure images has been requested from Cayman Chemical Company, Inc.

A. Resolvin D1

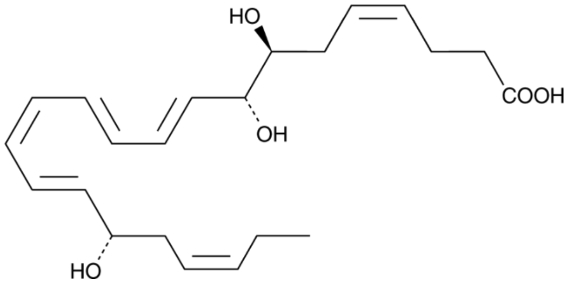

B. Protectin D1

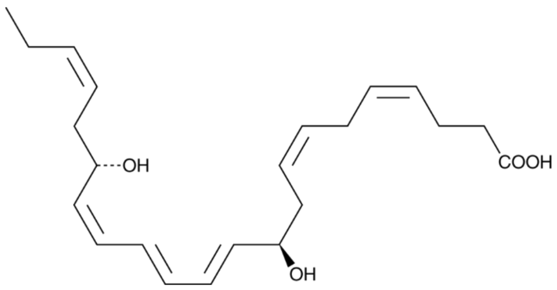

C. Maresin 1

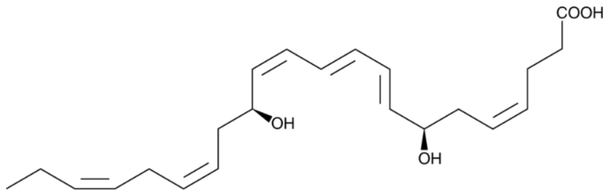

References

1. Cayman Chemical. *Resolvin D1*; Item No. 10012554. Available online: https://www.caymanchem.com/product/10012554/resolvin-d1 (accessed on 27 July 2026).
2. Cayman Chemical. *Protectin D1 (Neuroprotectin D1, NPD1, PD1)*; Item No. 10010390. Available online: https://www.caymanchem.com/product/10010390/protectin-d1 (accessed on 27 July 2026).
3. Cayman Chemical. *Maresin 1 (MaR1)*; Item No. 10878. Available online: https://www.caymanchem.com/product/10878/maresin-1 (accessed on 27 July 2026).
